# Assessment of the anterior segment of patients with primary congenital glaucoma using handheld optical coherence tomography

**DOI:** 10.1038/s41433-019-0369-3

**Published:** 2019-03-18

**Authors:** Anastasia V. Pilat, Frank A. Proudlock, Sonal Shah, Viral Sheth, Ravi Purohit, Joseph Abbot, Irene Gottlob

**Affiliations:** 1 Ophthalmology Group, University of Leicester, Leicester, UK; 2 Ophthalmology Department, Birmingham Children’s Hospital, Birmingham, UK

**Keywords:** Eye diseases, Pathogenesis

## Abstract

**Purpose:**

To investigate the potential of handheld optical coherence tomography (HH-OCT) in assessing the anterior segment of the eye in patients with primary congenital glaucoma.

**Design:**

A prospective, case-controlled observational study.

**Participants:**

Twenty-two patients with primary congenital glaucoma (PCG, 9 females and 13 males; mean age 4.36 ± 3.4 years) and age-, gender- and ethnicity-matched healthy participants.

**Methods:**

Anterior OCT was performed in all participants using a high-resolution HH SD-OCT device (Envisu 2300, Leica Microsystems, Germany) without anaesthesia or sedation.

**Results:**

Anterior HH-OCT in PCG visualised Haab’s striae in 14.3%, uneven internal cornea in 9.5% and epithelial thickening in 11.9% of patients with central corneal thickening (CCT, *p* < 0.001). CCT was significantly correlated with the intraocular pressure (IOP, *p* < 0.001). The flat iris with a thin collarette zone was found in 59.5%, anterior iris insertion in 11.90% of eyes affected by PCG. Two independent examiners showed sensitivity and specificity of 87% and 77%, respectively, by instating iris thinning and flattening of the anterior profile.

**Conclusions:**

Anterior HH-OCT has significant potential to improve diagnosis and management of PCG. Clinically relevant information can be obtained non-invasively and without sedation. High specificity makes anterior HH-OCT an important adjunct for management of PCG. Excellent visualisation of the iris insertion on OCT indicates potential for AS OCT to assist with surgical planning, including decision on the type of surgery and location of the incision.

## Introduction

From the 6th week of foetal development, anterior segment structures of the eye undergo a major transformation [1] with formation of the trabecular meshwork between 12 and 22 weeks and Schlemm’s canal at 16 weeks of age. Development is completed by 8 years of age [2]. Multiple structures are affected in different conditions associated with anterior angle maldevelopment in childhood glaucomas [3, 4]. Primary congenital glaucoma (PCG) is among one of the most common types of childhood glaucoma with a prevalence of over 32% [5].

Ko et al., suspected that the anterior chamber angles (ACA) appearance in PCG is a result from arrested neural crest tissues development with the suggested site of the outflow obstruction at the level of trabeculae [2, 6]. Rojas et al. investigated fragments of tissues after trabeculectomies in four patients with primary glaucoma and found high iris insertion and enlarged trabeculae with diminished inter-trabecular spaces with normal appearance of Schlemm’s canal [7]. In the earlier histopathology study, assessing ACA specimens after trabeculotomy and/or trabeculectomy (10 eyes), Anderson found that patients with PCG had thicker trabecular beams with both histologic and clinical evidence of iris root traction that obstructs the aqueous outflow. Anderson suggested that this is the result of the premature or excessive formation of the collagenous beams in the trabecular meshwork [8].

Prompt diagnosis of childhood glaucoma is essential to reduce the degree of visual impairment. Together with optic disc cupping, AS changes such as an increased corneal diameter and changes on gonioscopy are hallmarks for the diagnosis and management of childhood glaucoma [9]. However, examining and diagnosing young children is challenging as slit-lamp examination is not always possible, and elevated IOP can be variable and influenced by anaesthetic agents. Currently, several techniques for AS imaging are available, including slit scanning topography, ultrasound biomicroscopy, Scheimpflug and Purkinje imaging and optical coherence tomography (OCT) [10]. Recent studies demonstrate a wide clinical application range for OCT in AS imaging, including evaluation of the tear film and meniscus, contact lens fitting and assessing the cornea with pachymetric mapping and intraoperative OCT in children with congenital corneal opacities [11, 12].

OCT can also be used to monitor anterior chamber angle structures in adulthood glaucoma, including detecting the causes of angle closure and visualising the results of filtering surgery [13, 14].

Handheld OCT has recently become available and allows scans to be obtained in infants and small children who have previously been deprived of this technique. By contrast to adult glaucoma, the potential applications of OCT in childhood glaucoma are unknown. In this study, we investigated the potential of HH-OCT to improve the diagnosis of congenital primary glaucoma visualising the anterior eye structures without general anaesthetic or sedation.

Our aims were to investigate the potential of the HH-OCT in the anterior chamber assessment, including: (i) corneal thickness and internal contour of the endothelium; (ii) the structure of the iris and irido-corneal angle in PCG.

## Participants and methods

### Participants

This observational case control study included 22 patients with PCG (9 females and 13 males; mean age 4.26 ± 3.43), and 22 age-, gender- and ethnicity-matched healthy controls.

Patients were prospectively recruited from paediatric glaucoma clinics at Birmingham Children’s Hospital and from the University Hospitals of Leicester. The diagnosis of PCG was defined based on the 9th Consensus Report of the World Glaucoma Association (http://www.worldglaucoma.org/consensus-9/). The main diagnostic criteria included evidence of elevated IOP (raised IOP, enlarged horizontal corneal diameter, increasing axial length and progressive myopia), anomalous ACA on gonioscopy and glaucomatous optic neuropathy (increased cup/disc ratio, optic nerve cupping and no other syndromes/lenticular anomalies identified).

Only subjects with no known ophthalmic or general health pathology other than glaucoma were included. In addition, we imaged the iris of one patient with open-angle glaucoma (65-year-old female, Asian) and one premature infant (26 weeks of gestational age, male, Asian) for exploratory comparison with PCG.

The study adhered to the tenets of the Declaration of Helsinki and was approved by the local ethics committee. Informed consent was obtained from all participants or their parents or guardians.

For all patients, ophthalmic examination included best-corrected visual acuity, cycloplegic refraction, slit-lamp examination, horizontal corneal diameter measurements, fundus examination and measurements of IOP (i-care TAO1i, Finland/Goldman tonometry, if possible). In younger infants and children unable to cooperate with optotype visual acuity, visual acuity was assessed by preferential looking using Teller acuity cards. In cooperative participants, we used uncrowded or if possible crowded optotypes (Kay pictures) or Snellen test chart.

Supplementary Tables 1 (patients) and 2 (healthy controls) show the clinical and demographic characteristics of the participants of our study.

### Optical coherence tomography imaging

HH-OCT was performed without anaesthesia or sedation in outpatient clinics. A handheld SD-OCT device (Envisu 2300, Leica Microsystems, Germany), 840 -nm wavelength, 2.6 -mm theoretic axial resolution was used.

### Scan acquisition

Anterior segment imaging included one wide scan for assessing the cornea and anterior chamber (both angles, temporal and nasal) on the same image (18 × 6-mm horizontal scans with 3000 A scans and 11 B scans, 0.96 s of acquisition time). For detailed assessment of the angle structures, we used two independent smaller high-density scans for the temporal and nasal angle to obtain high-quality images, but simultaneously ensuring a short acquisition time important for optimising patient compliance (5 × 5-mm horizontal scans with 1200 A scans and 12 B scans, 0.44 s of acquisition time).

**Fig. 1:**
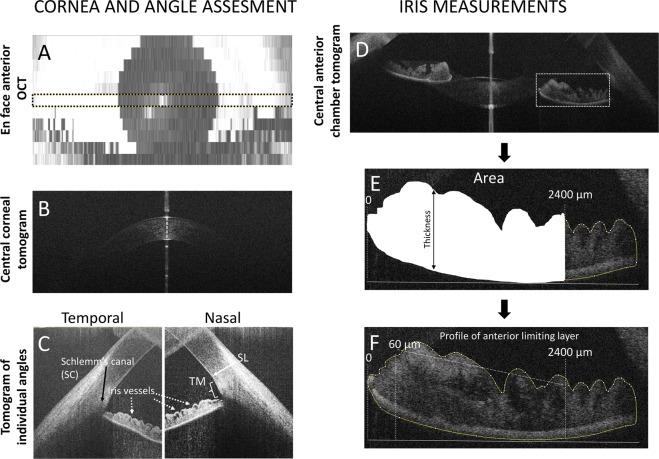
Optical coherence tomography (OCT) images of the anterior segment of the eye: **a** En face image showing the central position of the horizontal scan that was used for AS analysis (between dashed lines); **b** horizontal tomogram of the central cornea; **c** nasal and temporal high-resolution tomograms (1200 A scans and 12 B scans) indicating angle landmarks, including Schwalbe line (SL), trabecular meshwork (TM) and Schlemm’s canal (SC); **d** horizontal tomogram showing temporal and nasal irido-corneal angles; the iris area limited by anterior limiting membrane (ALM) and posterior epithelium (PE) was measured from the pupil to 2400 µm temporally from the pupil; the average iris thickness was measured between ALM and PE (**e**); standard deviation of the profile of ALM (**f**) was measured between 60 and 2400 µm temporally from the pupil

The en face scan shows the position of the anterior scan where the cornea and iris were analysed (Fig. 1). Qualitative analysis of the cornea included the assessment of the presence of endothelial changes, uneven contour of the cornea and change in the thickness of the epithelium and stroma (Fig. 1).

Assessment of the ACA structures (Fig. 1c) was based on the angle landmark location described by Bald et al. [15]. We evaluated the presence of the pathological anterior iris insertion and the visibility of the trabecular meshwork.

Anterior segment examination also included quantitative assessment of the thickness of the iris using imaged J, the averaged iris thickness/area were measured from the pupil to 2400 µm temporally/nasally (Fig. 1d–e) as the iris structure in glaucoma patients was different on visual inspection, as compared with healthy participants. Tortuosity of the profile of the iris anterior limiting membrane was measured by calculating the standard deviation of the anterior limiting membrane surface between 60 and 2400 µm from the pupil (Fig. 1f).

Two independent investigators were asked to judge for each patient by inspecting the anterior OCT scans whether they have glaucoma by deciding the presence of an abnormal thin iris and flattening of the anterior iris profile.

### Statistical analysis

Statistical analysis was performed using SPSS software version 16.0 (SPSS, Inc., Chicago, IL). Central corneal thickness and iris thickness parameters were normally distributed as determined by Shapiro–Wilk test. Quantitative parameters were analysed using a univariate mixed linear model, which included group as a fixed factor. Clinical factors (horizontal corneal diameter and central corneal thickness) were correlated using Pearson correlation; clinico-demographic parameters (corneal/iris parameters and the number of surgeries performed, clinical decision of the necessity of further surgery and age at examination) were correlated using Spearman’s coefficient of correlation. *P* *≤* 0.05 was considered statistically significant.

The sensitivity and specificity of PCG features detection on blind visual inspection of the iris tomograms by two independent investigators were calculated using the standard formulas. True positive results—PCG features found on OCT tomograms while patients had established PCG diagnosis based on clinical examination; true negative result—no PCG features found on OCT tomograms while patients did not have PCG based on previous data; false-positive result—PCG features found on OCT tomograms of healthy controls; false-negative result—no PCG features detected on OCT tomograms while patients had confirmed PCG.

## Results

### Anterior segment morphology

**Fig. 2:**
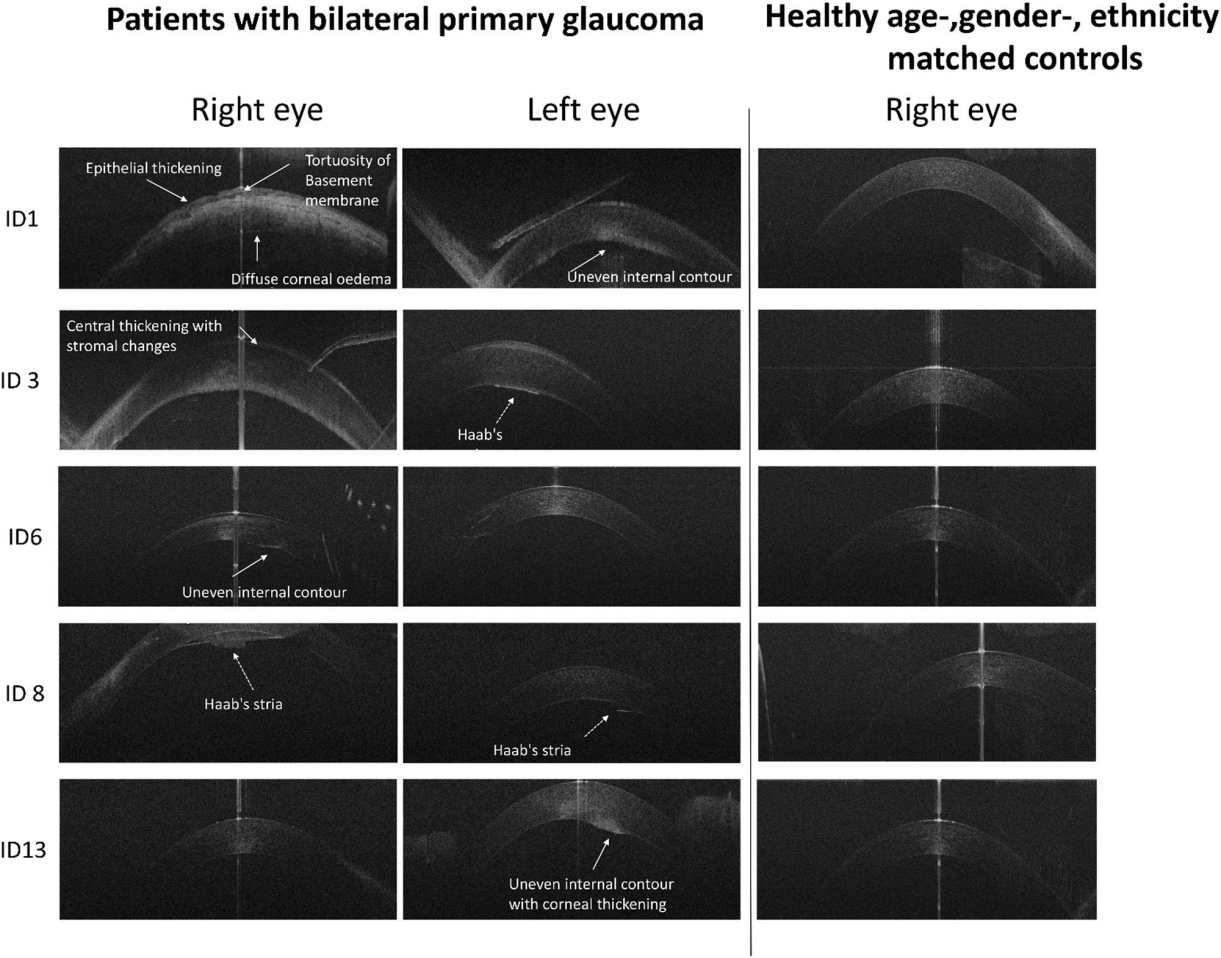
Corneal horizontal spectral domain–optical coherence tomography B-scan images of patients with PCG and healthy age-, gender- and ethnicity-matched controls. Patients with PCG showed a variety of changes, including epithelial thickening with a tortuous contour of the Bowman membrane (ID1, 6 and 13), uneven internal contour and the presence of Haab’s striae (ID3, 8)

Corneal assessment with anterior OCT was successful in 37 eyes (90.2% of eyes) with glaucoma. Haab’s striae were identified by the presence of an interrupted Descemet layer with a folded endothelium (Fig. 2, ID3 and 8). Visual inspection of the OCT scans in PCG showed the presence of Haab’s striae in 14.28% of the eyes. An uneven internal contour of the cornea was seen in 9.52% epithelial thickening with a tortuous profile of the Bowman’s layer in 11.9% of the eyes with PCG (Fig. 2, ID1 and 6). Uneven internal corneal contour presented only in patients with a clinically severe corneal oedema, who were awaiting further surgery.

The central cornea was significantly thicker in patients with PCG than in control children (453.59 ± 62.72 and 437.07 ± 30.84 µm for PCG and controls, respectively, *p* < 0.001) and showed a significant correlation with intraocular pressure at the time of OCT examination (IOP, *p* < 0.001). Horizontal corneal diameter, number of surgeries performed, clinical decision of the necessity of further surgery and age of the patients did not correlate with central corneal thickness. The central corneal thickness also significantly correlated with the necessity of further surgery (*p* < 0.001).

Visualisation and qualitative assessment of the irido-corneal angles was successful in 32 out of 44 eyes (76%). We were not able to assess the irido-corneal angle in eight eyes of four patients due to lack of cooperation and in four eyes of two patients because of nontransparent corneas.

**Fig. 3:**
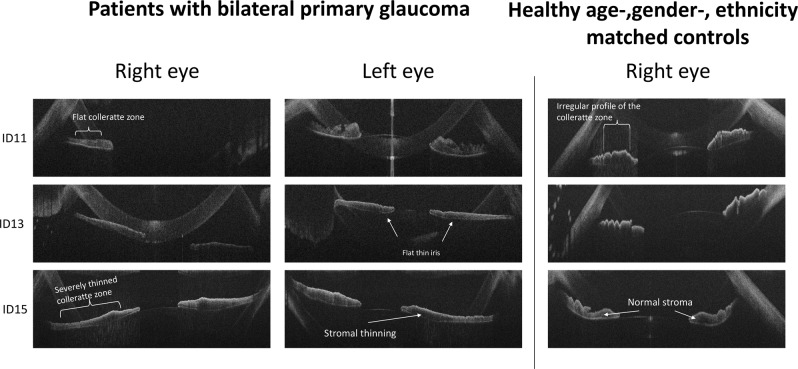
Horizontal spectral domain–optical coherence tomography B-scan images showing iris changes in patients with PCG and healthy age-, gender- and ethnicity-matched controls

Figure 3 shows different iris changes in patients with PCG as compared with healthy controls.

On visual inspection, a large variability of the iris structure was seen. A thinner iris with reduced iris folds due to reduced stromal thickness was found in 59.52% of patients in PCG.

**Fig. 4:**
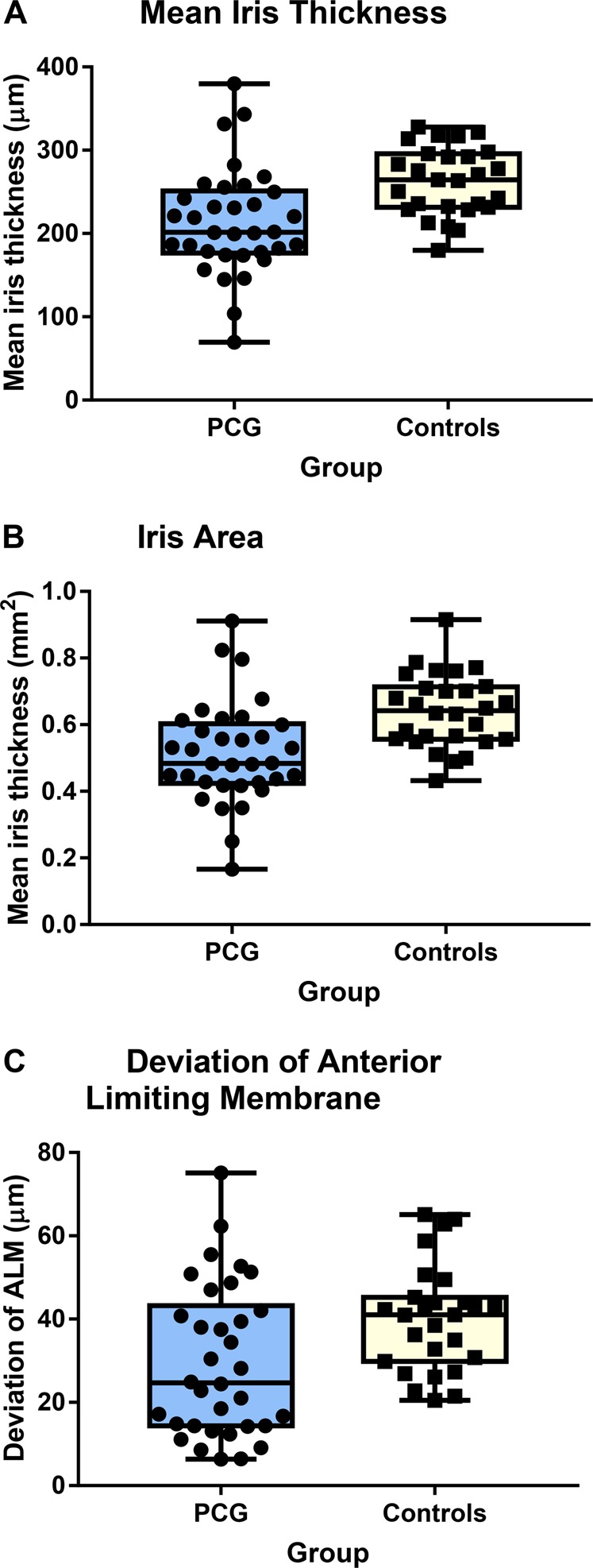
Distribution and mean ± SD of (**a**) iris thickness, (**b**) area and (**c**) deviation of the anterior limiting membrane in patients with primary congenital glaucoma (PCG) and healthy controls

Statistical analysis showed a reduced iris thickness (213.67 ± 63.26 and 267.24 ± 45.85 µm for PCG and controls, respectively, *p* < 0.001) and reduced iris area (512,836 ± 151,831 and 641,389 ± 110,043 µm^2^ for PCC and controls, respectively, *p* < 0.001) in the PCG group. Patients with PCG also had a severe flattening of the anterior limiting layer with a significant larger standard deviation in the control group (29.54 and 42.75 for PCG and controls, respectively, *p* = 0.006). Figure 4 shows individual values for the iris parameters.

Averaged sensitivity and specificity based on the iris thinning and flattening of the anterior limiting membranes on the visual inspection of the scans of patients with PCG and controls by the two independent investigators were 0.87 ± 0.02 and 0.77 ± 0.03, respectively.

**Fig. 5:**
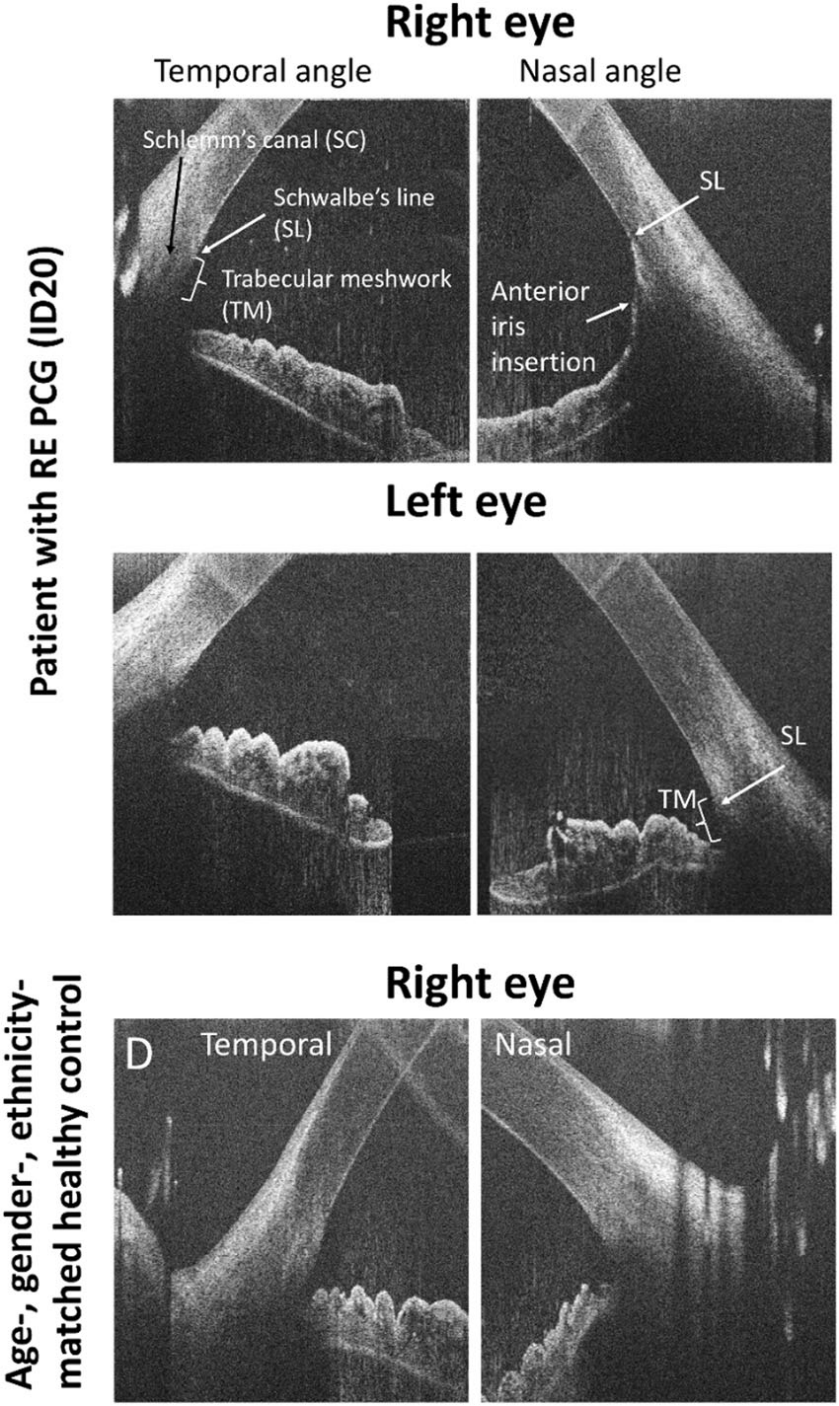
Horizontal high-resolution spectral domain–optical coherence tomography B-scan images of the temporal and nasal irido-corneal angles in a patient with PCG in the right eye and a healthy age-, gender- and ethnicity-matched control. The image of the right affected eye of the patient shows abnormal anterior iris insertion in the nasal angle with the iris rout inserting at Schwalbe line (SL) covering the trabecular meshwork (TM). Normal configuration with a visible trabecular meshwork in the temporal angle of the affected eye, in the non-affected eye as well as in the control subject

Abnormal iris insertion in front of the SL (Fig. 5) was detected in 11.90% of affected eyes on the single horizontal tomogram.

We did not find any significant correlations between the mean iris thickness and area with clinical or demographic parameters.

## Discussion

This study investigates the potential of the HH-OCT in the assessment of the anterior segment structures in patients with PCG. Not all the parameters assessed could be used and will be used clinically. However, the study provides additional information for the understanding of the structural changes in the anterior segment due to raised intraocular pressure in young children.

In this study, assessment of structural changes of the anterior segment was successful in 90% for the cornea and 76% for the anterior angle of the patients with PCG using HH-OCT without anaesthesia. The main findings included Haab’s striae, uneven internal contour and epithelial thickening of the cornea in 36% of eyes. In our study, the occurrence of Haab’s striae (14.28%) was less frequent than that observed by Patil et al. who found Haab’s striae in 44.8% of eyes with PCG using OCT [16]. Differences could be explained by older age of participants and hence a longer duration of disease in the study by Patil et al. In addition, in our study, the assessment was performed on one single horizontal scan, whereas Patil et al. have assessed a larger corneal area.

Thickening of the cornea was found in several other studies using pachimetry [17–19] and UBM [20]. In our PCG patients, central corneal thickness was also significantly increased with a positive correlation with increased IOP. The cornea was thicker in patients with poor IOP control awaiting surgery. In agreement with Cronemberger et al. [17], this indicates that increased central corneal thickness is likely to be associated with decompensated pressure and need for further treatment.

Our data showed significant thinner iris and flattening of the anterior limiting layer in PCG. These parameters were highly sensitive and specific for PCG distinguishing from control patients by inspection of two independent examiners (0.87 ± 0.02 and 0.77 ± 0.03, respectively). Hussain et al. also found iris thinning measured at 2 mm from the pupil in patients with PCG using ultrasound biomicroscopy (UBM); however, the data were not significantly different from controls [20]. This could be explained by a higher sensitivity of the parameters (mean thickness/area) used in our study rather than single thickness measurement. Using UBM, Gupta et al. found significant negative correlation between iris thickness and axial length in PCG [21] and, therefore, hypothesised the presence of iris ‘stretching' with increased axial length. The cause of iris thinning is not known. Corneal diameter increases with high IOP due to stretching of the eye [17]. In our study, there was no correlation between the iris area/thickness and raised IOP or horizontal corneal diameter. This argues against the iris being flat due to stretching of the eye related to increased IOP. For comparison, we have analysed the iris structure of an adult with OAG and have not found an association with thinning of the iris or an abnormal irregular profile of the iris anterior limiting layer (Supplementary Figure 1). Therefore, elevated IOP may not play a significant role in iris thinning. However, the iris structures may be more susceptible to damage due to elevated IOP in young children similarly to other structures of the eye.

Another possibility could be that the development of iris structures is prevented after birth due to IOP elevation early in life in PCG. However, in premature babies (example shown in Supplementary Figure 1), we have observed already at the 26th gestational week a well-formed iris structure with a tortuous profile of the anterior limiting layer similar to older subjects. Therefore, iris thinning and other abnormal anterior segment changes, such as anterior iris insertion, are likely to be part of a primary maldevelopment of the AS in PCG. Similarly, a large range of anterior chamber phenotypes have been described within families with identical genetic mutations in anterior segment dysgenesis, such as Axenfeld’s, Rieger’s, Peters’ anomalies, aniridia or PCG [22].

In adult angle-closure glaucoma, Lai et al. described good visualisation of the structures of the angle with swept-source OCT and showed that OCT is useful in assessing the risk of angle closure [23–25]. In our study on the single horizontal OCT scan, iris insertion was clearly visualised in 98% of patients showing excellent feasibility. Abnormal anterior iris insertion on a single horizontal scan was found in 12% of PCG patients. Hussein et al. reported anterior iris insertion in 56% eyes with PCG using UBM in older children. UBM allows 360° angle assessment likely to explain a higher detection rate [20].

In our study, we found OCT to be a useful technique to add essential information for a clinician. The presence of increased corneal thickness on OCT was a sign of reduced pressure control. It did not correlate with the corneal diameter; however, it did correlate significantly with IOP measured on the day of the examination. It was also possible to perform additional noninvasive objective measurements using OCT, including a horizontal corneal diameter, visualisation of Haab’s striae and angle assessments, when gonioscopy would be difficult in young patients in the clinic.

On visual inspection, the image quality and detailing of the anterior angle structures on OCT scan appears better than that shown using UBM [20, 21]. A clear visualisation of anterior iris insertion on OCT supports the potential of OCT to be a powerful technique to assess the anterior angle and to add information or possibly even substitute gonioscopy in surgical planning. OCT could be used to determine the localisation of the goniotomy or trabeculotomy and to monitor the effectiveness of the surgical treatment. OCT has the potential to be used intraoperatively to find the segments most affected by the anterior position of the iris, to monitor the procedure by control of localisation and depth of the incision, determination of the position of the tube shunts or valves, etc.

A limitation of our study was that we only assessed the iris insertion horizontally. Further studies are needed to develop a strategy for angle assessment at several locations. Vertical and oblique single scans could be used to assess the angle at various locations; however, limited time available for maintaining cooperation of young children makes acquisition of multiple scans difficult. Current techniques using the handheld OCT in young children who do not have stable fixation during image acquisition only allow multiple single scans limiting use of the radial 360° scans centred on the cornea or the centre of the pupil due to poor fixation. However, 360° scans could be obtained preoperatively under anaesthesia as these are performed with intraoperative OCT in children [12]. In this study, we did not aim to investigate the reproducibility of the findings and clinical relevance of the OCT data. The main idea of this work was to show that OCT can be used without anaesthesia and sedation in the normal clinical surrounding, and the cornea, angles and iris structure could be well seen in children who cannot cooperate with table-mounted devices. Therefore, further work with longitudinal data is required to assess the clinical significance of the findings and reproducibility of the assessment.

In conclusion, anterior HH-OCT has a significant potential to improve the diagnosis and management of PCG. Important clinically relevant information helping in the diagnosis and management can be obtained non-invasively without GA or sedation. Prompt diagnosis of PCG is essential for limiting visual impairment. In addition to the assessment of optic nerve changes and elevated IOP, diagnosis is based on anterior segment examination. However, diagnosis is difficult and limited by the child’s cooperation with the slit lamp. We have shown a high specificity of the diagnosis of glaucoma using anterior segment OCT which provides high resolution, stable visualisation and quantitative documentation of the changes for follow-up.

### Summary

#### What was known before

- The pathogenesis of PCG is unclear.
- Immature development of the anterior chamber is likely to be the main cause of elevated intraocular pressure.
- Anterior OCT can give important information.
- Infants and small children are deprived of the anterior segment OCT.

#### What this study adds

- Anterior HH-OCT has significant potential to improve the diagnosis and management of PCG.
- Clinically relevant information can be obtained non-invasively and without sedation.
- Excellent visualisation of the iris insertion on OCT indicates potential for AS OCT to assist with surgical planning.

## Supplementary information

Supplementary table 1

Supplementary table 2

Supplementary figure legend

Supplementary figure 1
