# Supplementary material for: Assessment of the anterior segment of patients with primary congenital glaucoma using handheld optical coherence tomography
Source: Eye (Lond). 2019 Mar 18;33(8):1232–9. doi: 10.1038/s41433-019-0369-3 (PMC7005739; doi:10.1038/s41433-019-0369-3)
Supplement: Supplementary file 1 — Supplementary table 1 [file 41433_2019_369_MOESM1_ESM.docx]

**Supplementary table 1:** Clinical and demographic characteristics of the patients with primary congenital glaucoma/open angle glaucoma who participated in the study.


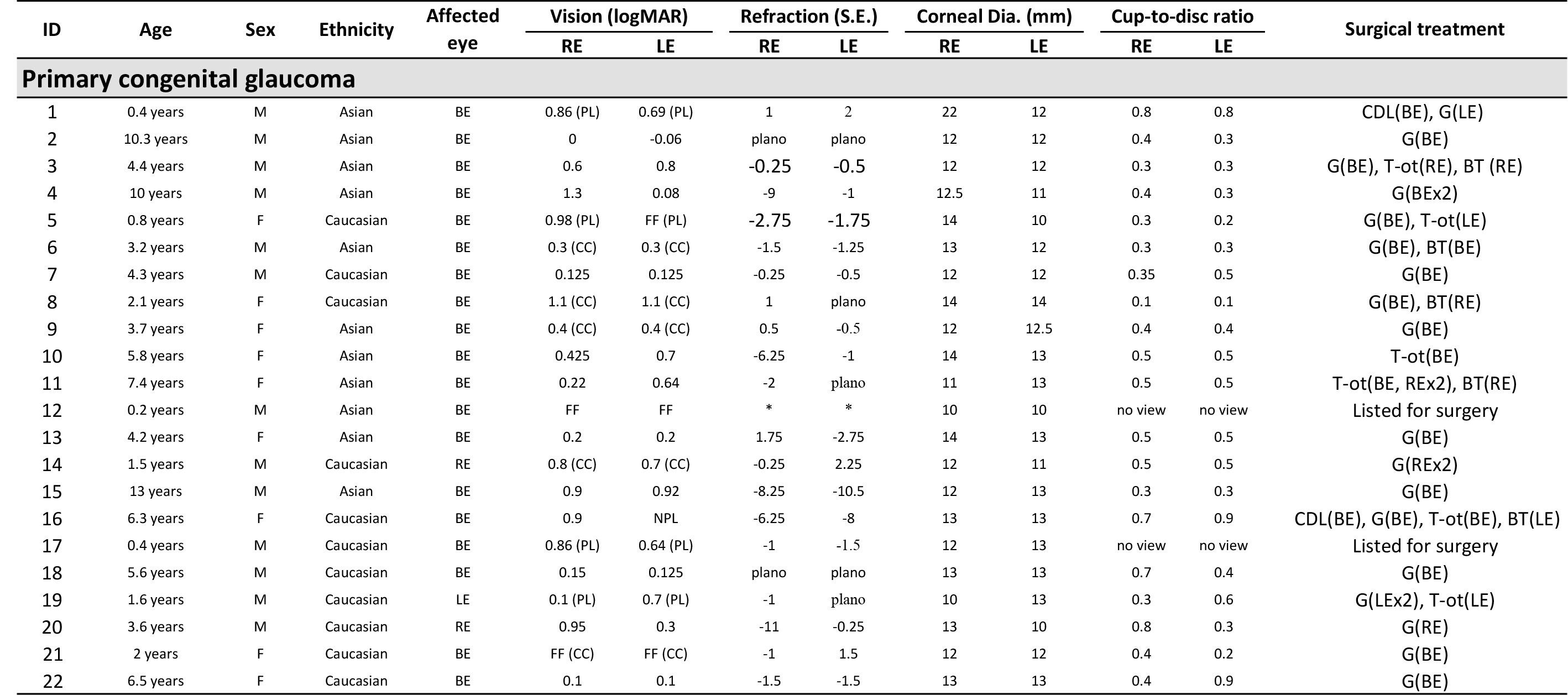


M=male, F=female; RE=right eye, LE=left eye, BE=both eyes; S.E. = spherical equivalent; FF=following and fixing; PL= preferential looking; CC=Cardiff cards; in all other participants Kay pictures or Snellen test were used, NPL=no perception of light, G=goniotomy, T-ot=Trabeculotomy, BT=Baelvedt tube, CDL=cyclodiode laser.
