# Supplementary material for: Assessment of the anterior segment of patients with primary congenital glaucoma using handheld optical coherence tomography
Source: Eye (Lond). 2019 Mar 18;33(8):1232–9. doi: 10.1038/s41433-019-0369-3 (PMC7005739; doi:10.1038/s41433-019-0369-3)
Supplement: Supplementary file 3 — Supplementary figure legend [file 41433_2019_369_MOESM3_ESM.docx]

***Supplementary figure 1:*** *Central horizontal spectral domain-optical coherence tomography B-scan images (right eye) showing irides and irido-cornel angles in patients with PCG, OAG and healthy age-, gender-, ethnicity matched controls and a 26 weeks gestational age premature infant (M=male, A=Asian). The patient with PCG has a flat and thin iris while in the other participants the anterior limiting membrane profile is “tortuous” with a thicker collarette zone.*
