# Supplementary figures and images for: Assessment of the anterior segment of patients with primary congenital glaucoma using handheld optical coherence tomography
Source: Eye (Lond). 2019 Mar 18;33(8):1232–9. doi: 10.1038/s41433-019-0369-3 (PMC7005739; doi:10.1038/s41433-019-0369-3)

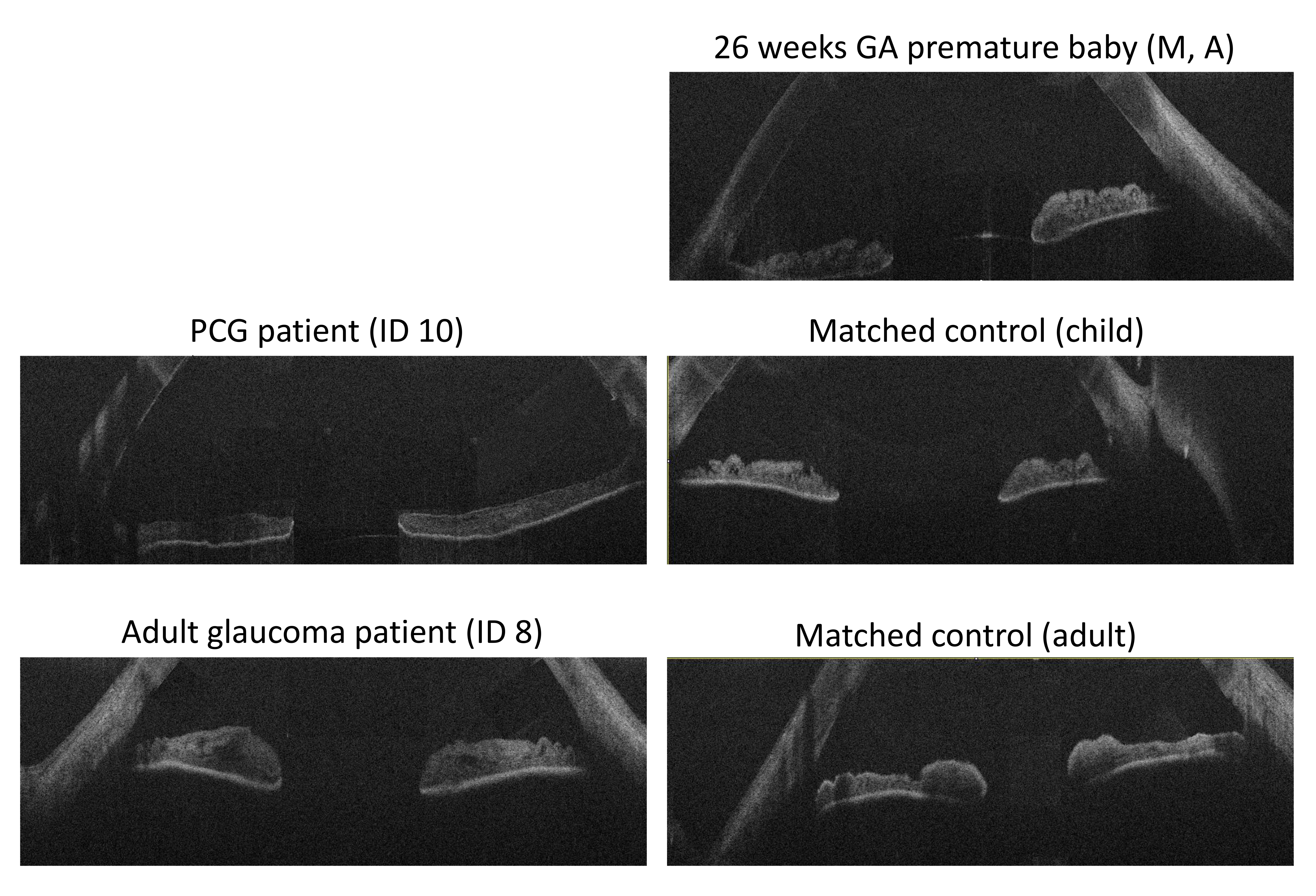

Supplement: Supplementary file 4 — Supplementary figure 1 [file 41433_2019_369_MOESM4_ESM.tif]
